# Photoreduction of Carbon Dioxide to Formic Acid in Aqueous Suspension: A Comparison between Phthalocyanine/TiO_2_ and Porphyrin/TiO_2_ Catalysed Processes

**DOI:** 10.3390/molecules20010396

**Published:** 2014-12-30

**Authors:** Giuseppe Mele, Cosimo Annese, Lucia D’Accolti, Alberto De Riccardis, Caterina Fusco, Leonardo Palmisano, Anna Scarlino, Giuseppe Vasapollo

**Affiliations:** 1 Department of Engineering for Innovation, University of Salento, via Arnesano, Lecce 73100, Italy; E-Mails: alberto.dericcardis@unisalento.it (A.D.R.); anna.scarlino@unisalento.it (A.S.); giuseppe.vasapollo@unisalento.it (G.V.); 2 CNR-ICCOM UOS of Bari, Chemistry Department, University of Bari, Via Orabona, 4, Bari 70126, Italy; E-Mails: cosimo.annese@uniba.it (C.A.); fusco@ba.iccom.cnr.it (C.F.); 3 Chemistry Department, University of Bari “A. Moro”, via Orabona, 4, Bari 70126, Italy; E-Mail: lucia.daccolti@uniba.it; 4 Dipartimento di Energia, Ingegneria dell’Informazione e Modelli Matematici, DEIM, Università di Palermo, Viale delle Scienze, Ed. 6, Palermo 90128, Italy; E-Mail: leonardo.palmisano@unipa.it

**Keywords:** CO_2_, heterogeneous photocatalysis, phthalocyanines, porphyrins, photo-reduction, TiO_2_

## Abstract

Composite materials prepared by loading polycrystalline TiO_2_ powders with lipophilic highly branched Cu(II)- and metal-free phthalocyanines or porphyrins, which have been used in the past as photocatalysts for photodegradative processes, have been successfully tested for the efficient photoreduction of carbon dioxide in aqueous suspension affording significant amounts of formic acid. The results indicated that the presence of the sensitizers is beneficial for the photoactivity, confirming the important role of Cu(II) co-ordinated in the middle of the macrocycles. A comparison between Cu(II) phthalocyanines and Cu(II) porphyrins indicated that the Cu(II)- phthalocyanine sensitizer was more efficient in the photoreduction of CO_2_ to formic acid, probably due to its favorable reduction potential.

## 1. Introduction

Carbon dioxide (CO_2_) is a well-known greenhouse gas which can be emitted to the environment by natural sources as well as by human activities. It could be considered one of the simplest alternative feedstocks, and has thus attracted the interest of industrial and academic researchers due to its low cost and worldwide availability.

The production of chemicals from CO_2_ would not only preserve the oil reserves but also would reduce the CO_2_ emissions [1,2]. As it is well known, CO_2_ is extremely thermodynamically stable, and for this reason its reduction is a difficult task.

Some progress has been made to enhance the yield of the reduction by means of various strategies, e.g., by reducing CO_2_ with H_2_ in supercritical fluids [3]. The catalytic activation of CO_2_, however, has been the preferred approach in recent years. In particular the synthesis of formic acid (HCOOH) from CO_2_ has reached such a state of knowledge that the continued progress of this research may well lead to good practices the treatment of CO_2_ produced on an industrial scale. A fruitful interaction between investigative work into the reaction mechanism(s) and the development of new catalytic systems would therefore seem desirable [4].

Inoue and co-workers reported pioneering studies concerning the photocatalytic reduction of CO_2_ in aqueous solution to produce HCOOH, along with other organic molecules, using various semiconductors including titanium dioxide (TiO_2_) [5].

Photocatalytic reduction of CO_2_ using photosensitive semiconductor powders has been widely studied in aqueous solutions [6,7,8,9,10,11,12,13,14,15,16,17,18,19,20,21,22] or supercritical fluid [23]. In particular, the photoreduction of CO_2_ with water into organic compounds using various TiO_2_ photocatalysts has attracted the major interest.

TiO_2_ is one of the most widely used photocatalysts in the field of environmental applications due to its low toxicity, ability to resist to photocorrosion, and low cost. However, the large band gap of titania and massive recombination of photogenerated charge carriers limit its overall photocatalytic efficiency.

These problems could be overcome by modifying the electronic band structure of TiO_2_, which is possible using various strategies like coupling with a narrow band gap semiconductor, metal ion/nonmetal ion doping, co-doping with two or more foreign ions, surface sensitization by organic dyes or metal complexes, and noble metal deposition [2,24].

The presence of suitable modifiers is important in order to perform the photoreduction processes occurring under visible or solar light radiation [25,26,27]. For example CO_2_ was photocatalytically reduced to produce methanol by using TiO_2_ and Cu-TiO_2_ in aqueous suspension under UV irradiation [28]. In other studies an efficient and clean photoreduction to CO was obtained by using enzyme-modified TiO_2_ nanoparticles under visible light [29].

An alternative strategy might be the use of sensitizers, in order to use visible radiation for the activation of TiO_2_. Phthalocyanine metalloderivatives (MPcs) or their supramolecular arrangements have recently attracted an increasing interest because they have been used not only as the main species for the preparation of dyes and pigments, but also as the building blocks for the construction of new molecular materials used in various technological applications [30,31,32], including some catalytic ones [33].

In the last years, dye-sensitized TiO_2_ processes have been employed for solar energy conversion, photocatalysis and (electro)-photography. These technologies can be actually considered affordable because of their relatively low potential costs, low environmental impact, and efficient power conversion [34,35]. The photocatalytic reduction of CO_2_ under anaerobic conditions, promoted by cobalt and iron phthalocyanines, for instance, produced CO and formate in DMF and acetonitrile solutions [36].

The combination of TiO_2_ with porphyrins (Pps), metal porphyrins (MPps), phthalocyanines (Pcs), metal phthalocyanines (MPcs) or other structurally similar species, allows one to obtain extremely versatile composite sensitized materials with not only oxidizing properties suitable for photodegrading organic pollutants in water [37,38,39], but also reductive properties to photoreduce CO_2_ [40,41].

Recently, the photoreduction of CO_2_ to formic acid in aqueous solution using cobalt (CoPc) and zinc phthalocyanine (ZnPc)-loaded TiO_2_ synthesized under visible light irradiation has been reported; the quantification of formic acid was performed by UV-Vis spectroscopy [42,43].

The main aim of this work was to compare hybrid photocatalysts, obtained by impregnation of sensitizers (Pcs and Pps) onto the TiO_2_ surface, previously used successfully for photodegradation reactions [37,38], and to demonstrate how some of them are suitable for an efficient photocatalytic reduction of CO_2_ to formic acid. In addition a mechanism is proposed for the role of sensitizer, based on the reduction potential values of the macrocycles.

**Figure 1:**
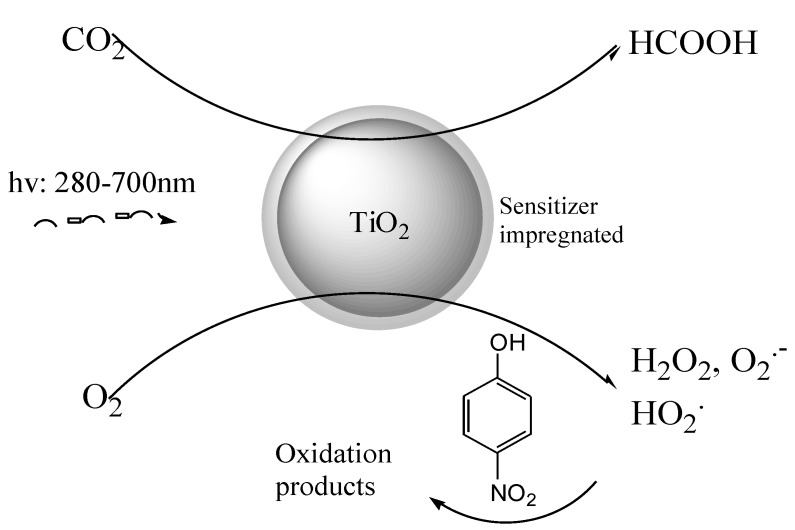
Double activity of CuPc-TiO_2_.

This new class of multipurpose materials (Figure 1) can be actually considered, due to their relatively low potential cost and the efficient power conversion. Their use has a low environmental impact and employs new reaction conditions: in fact the use of organic hole scavengers, previously reported in formic acid photosynthesis from CO_2_ [44] and also the use of strong alkaline pH values [42,43] is avoided.

## 2. Results and Discussion

### 2.1. Structures of the Sensitizers (MPcs, and MPps)

**Figure 2:**
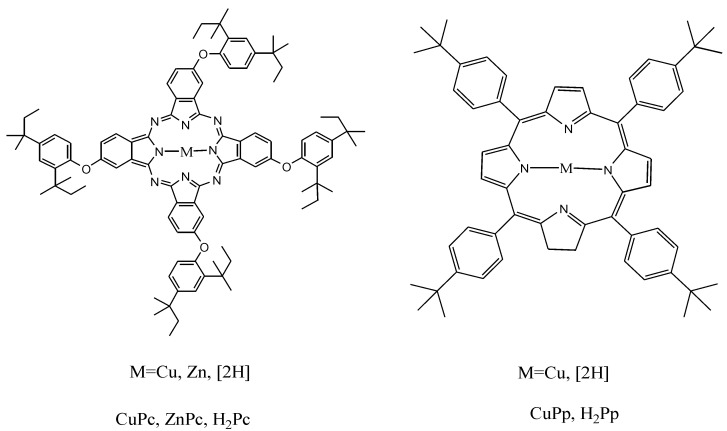
Molecular structure of MPc*s* and MPps.

The molecular structures of the metal free and metallo-porhyrins and phthalocyanines used as sensitizers in this work are reported in Figure 2. These compounds were opportunely synthesized, characterized and loaded onto TiO_2_ as described in the Experimental Section.

### 2.2. Photoreactivity Experiments

The photoreduction of CO_2_ to formic acid was chosen as a model reaction in order to evaluate the photocatalytic capabilities of the Pcs/TiO_2_ and Pps/TiO_2_ composites in comparison with the pristine TiO_2_. Three blank tests were carried out, according to the conditions of Process A (see Experimental Section, 3.6.1), in order to ensure that formic acid production derived only from the photoreduction of CO_2_. The first was carried out by irradiating the reaction system in the absence of catalyst while the second one was run under dark in the presence of catalyst. The third test was performed finally in the absence of CO_2_ to ensure that the formic acid was not obtained from some other ubiquitous organic species. No hydrocarbon was detected in the above blank tests. Further investigations were carried out in order to establish if the Pps or Pcs supported onto TiO_2_ were photostable, *i.e.*, if some decomposition of the supported sensitizers occurred under the same conditions used during the photocatalytic experiments.

No significant release of organic degradation sensitizers was observed even after long irradiation times (5–7 h) of the supported sensitizers used and also the sensitizer itself can be recovered quantitatively from the TiO_2_ surface by extraction with chloroform or dichloromethane. The absence of structural modifications for the phthalocyanine and porphyrins recovered was confirmed by the analytical and spectral data.

The use of ion chromatography to detect formic acid allowed its good separation from the interfering species. Quantification of formic acid by UV-Vis spectroscopy is very difficult, in fact, due to the interference of bicarbonate ions also at very acidic pHs. Optimization of the reaction conditions was performed when CuPc was used as sensitizer. Notably HCOOH was formed when both NaHCO_3_ and NaHCO_3_^13^C labelled were used.

A preliminary ^13^C GC/MS study on formic acid formation was performed using ^13^C enriched NaHCO_3_ as described previously (Process B, see Experimental Section, 3.6.2). First, 99% enriched CO_2_ was generated *in situ* from labelled bicarbonate by addition of phosphoric acid, then the reaction mixture was irradiated for 8 h.

**Figure 3:**
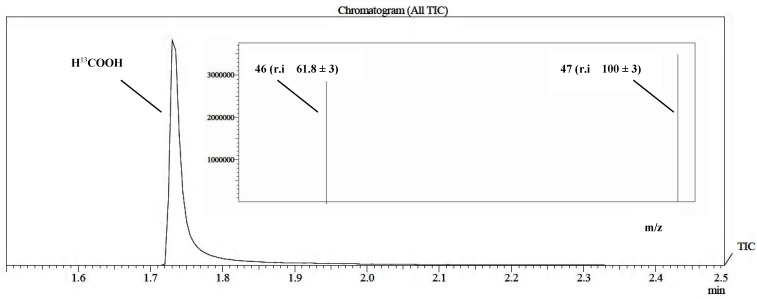
Qualitative determination of formic acid performed by GC/MS in SIM Mode analyzing an authentic sample of formic acid-^13^C 95 wt. % in H_2_O, 99 atom % ^13^C.

**Figure 4:**
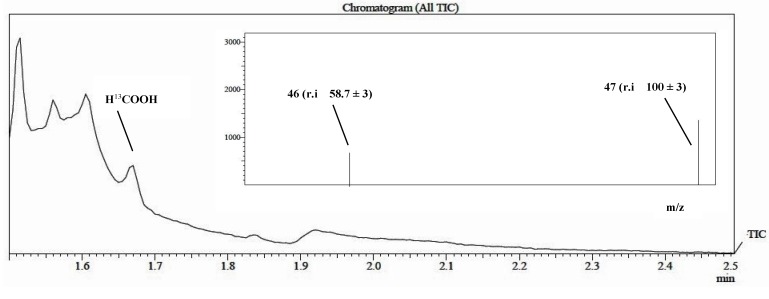
Qualitative determination of formic acid performed by GC/MS in SIM Mode analyzing formic acid-^13^C 9obtained by photoreduction of NaHCO_3_^13^C labelled.

The use of this particular source of CO_2_ allowed us only to qualitatively detect the formation of the product. The comparison with an authentic sample of labeled formic acid indicated that the produced formic acid derived from ^13^CO_2_ (Figure 3 and Figure 4). The reaction was monitored using the GC/MS in SIM mode acquisition, in this case the main advantage was the increase of the sensitivity of the instrument with respect to the acquisition in “full scan” acquisition.

### 2.3. Effect of the Amount of Catalyst

In order to determine the effect of the catalyst amount, a set of experiments was carried out by varying the amount of the 6.65-TiO_2_-CuPc photocatalysts in the range 0.025–0.300 g. The influence of the photocatalyst quantity on formic acid formation is reported in Table 1. It can be observed that 0.025 g represents the amount of catalyst producing the most significant increase of formic acid production if it is compared with bare TiO_2_ or higher loadings (0.075–0.300 g). Nevertheless a higher quantity, *i.e.*, 0.050 g, was used in the further experiments to avoid difficulties to reproduce the measurements. In the presence of 0.050 g of CuPc-TiO_2_ sample the yield of formic acid reached the value of 208.5 µmol/g_cat_ after 8 h irradiation, and this figure is close to that obtained by using 0.025 g of catalysts. The value obtained with 0.050 g is higher, however, than those obtained by Ishitani * et al.* [16], Hirano *et al.* [17] and Kaneco [23].

**Table 1:** Effect of photocatalyst amount on formic acid production efficiency at pH = 3.

Catalyst ^a^	g of Catalyst	mg/L *(g_cat_)^−1^^b^	μmol/g_cat_
TiO_2_-CuPc	0.300	34.6	37.6
TiO_2_-CuPc	0.150	36.0	39.2
TiO_2_-CuPc	0.075	83.4	90.6
TiO_2_-CuPc	0.050	191.8	208.5
TiO_2_-CuPc	0.025	220.4	239.5
TiO_2_	0.050	120.5	131.0
TiO_2_	0.025	169.9	184.7
TiO_2_^c^	0.050	-	-
TiO_2_-CuPc ^c^	0.050	-	-

^a^ Quantity of sensitizer = 6.65 μmol/g TiO_2_; ^b^ Irradiation time 8 h; ^c^ Reaction carried out in the absence of CO_2_.

### 2.4. Effect of the Initial pH

The influence of pH of the aqueous solution on the process efficiency for the formation of formic acid was studied by using three different initial pH values (3, 7 and 13) without changing or checking them throughout the experiments. In Table 2, the results of CO_2_ photoreduction at different initial pH’s have been reported. The conversion of CO_2_ into formic acid increased by decreasing the pH value. The highest conversion (CO_2_ to HCOOH) observed in this work was 0.65% and this value is reasonable when compared with others reported in the literature (0.2%–0.4%) [42].

**Table 2:** Effect of initial pH on the production efficiency of formic acid.

Catalyst ^a,b^	pH	mg/L *(g_cat_)^−1^ ^c^	μmol/g_cat_
TiO_2_-CuPc	13	60.0	65.2
TiO_2_-CuPc	7	58.3	63.4
TiO_2_-CuPc	3	191.8	208.5
TiO_2_	3	120.5	131.0

^a^ Quantity of sensitizer = 6.65 μmol/g TiO_2_; ^b^ Quantity of catalyst = 0.050 g; ^c^ Irradiation time 8 h.

It was calculated as ratio of the concentration of formic acid and the concentration of carbon dioxide in water by assuming the solution saturated by CO_2_ and by considering the relative constant of solubility equal to 1.45 g/L at 298 K. The hydration equilibrium at pH = 3 was negligible, being the constant of hydration equal to 1.70 × 10^−3^ at 298 K. This finding can be explained by considering both the role of hole scavengers and the reaction mechanism.

It has been widely reported [45,46,47,48,49] that the presence of hole scavengers enhances the photoactivity of TiO_2_. The role of scavengers is to prevent the hole-electron recombination and the interaction of *h^+^* with precursor(s) of the desired product.

Under almost neutral or strongly alkaline conditions CO_2_ can be more easily converted into HCO_3_^−^ and CO_3_^2−^ (see Equations (1) and (2)):

(1) $$CO_{2}+H_{2}O⇄\left[H_{2}CO_{3}\right]⇄HCO_{3}^{-}+H^{+}$$

(2) $$HCO_{3}^{-}⇄CO_{3}^{2-}+H^{+}$$

and both species are able to act as hole scavengers according to the following reactions:

HCO_3_^−^ + *h^+^* → HCO_3_^•^ (3)

CO_3_^2−^ + *h^+^* → CO_3_^•−^ (4)

By taking into account that the amounts of HCO_3_^−^ and CO_3_^2−^ anions, deriving from CO_2_, can be considered negligible in acidic solution, their role as radical scavengers can be considered negligible at the beginning of the process. However, their involvement throughout the process cannot be excluded, at least when an increase of pH occurred.

The increased amount of formic acid observed in the case of the acidic solutions may be also ascribed to protonation-deprotonation equilibria involving carbon dioxide or other related species which have been reported to induce desorption of the product from the surface of TiO_2_, also influencing the photoactivity. It is likely that the positive charge on the carbon atom as well as its formation close to the surface of the catalyst facilitates the reduction.

### 2.5. Effect of Copper in the CuPc-TiO_2_ Composite

CuPc-TiO_2_ sample showed the highest efficiency when compared to pure TiO_2_, H_2_Pc and ZnPc samples (Table 3).

**Table 3:** Effect of metal on formic acid production efficiency at pH = 3.

Catalyst ^a,b^	mg/L *(g_cat_)^−1^ ^c^	μmol/g_cat_
TiO_2_-CuPc	191.8	208.5
TiO_2_-H_2_Pc	69.0	75.0
TiO_2_-ZnPc	81.5	88.5
TiO_2_	120.5	131.0

^a^ Quantity of sensitizer = 6.65 μmol/g TiO_2_; ^b^ Quantity of catalyst = 0.050 g; ^c^ Irradiation time 8 h.

The better activity of CuPc could be ascribed to the high capacity of Cu^2+^ to be reduced to Cu^+^ and to transfer one electron to the [H-O-C=O]**^.^**^+^species.

A few years ago some of us demonstrated that the presence of copper coordinated in the middle of the macrocycle could play an important role in photo-oxidation reactions, favouring the formation of oxidant radical OH species [38,39], e.g., Equation (5):

TiO_2_[Cu(I)Sens] + ^3^O_2_ → TiO_2_[Cu(II)Sens] + ^•^O_2_^−^ (5)

Analogously, CO_2_ can be reduced by Cu(I) species present on the surface of TiO_2_ (see Equation (6)) producing the radical species hypothesized to be the precursor of HCOOH:

TiO_2_[Cu(I)Sens] + CO_2_ → TiO_2_[Cu(II)Sens] + CO_2_^•−^ (6)

However, the electron transfer involving the protonated CO_2_ species generated in the acidic media cannot be excluded *a priori*.

### 2.6. Effect of the Irradiation Source

The effect of irradiation source on the formic acid formation was investigated to select optimal irradiation conditions. The results are reported in Table 4.

**Table 4:** Effect of irradiation sources on formic acid production efficiency at pH = 3.

Catalyst ^a,b^	Irradiation Source	mg/L *(g_cat_)^−1^ ^c^	μmol/g_cat_
TiO_2_-CuPc	Sanolux (UV/Vis) Halogen (Vis)	191.8	208.5
TiO_2_	Sanolux (UV/Vis) Halogen (Vis)	120.5	131.0
TiO_2_-CuPc	Sanolux (UV/Vis)	30.0	32.6
TiO_2_-CuPc	Halogen (Vis)	48.0	52.2

^a^ Quantity of sensitizer = 6.65 μmol/g TiO_2_; ^b^ Quantity of catalyst = 0.050 g; ^c^ Irradiation time 8 h.

It can be seen that after 8 h the yield of formic acid increased when two irradiation sources were used simultaneously. On the contrary, a negligible photoactivity was observed when a single lamp was used as the irradiation source. Therefore, analogously to what observed for the photodegradation of 4-nitrophenol (4-NP) [37,38,39] it is essential to photoexcite both the components of the system, *i.e.*, TiO_2_ and the sensitizer, in order to obtain substantial improvement of the photoreduction of CO_2_. The beneficial effect on the photoreactivity is due probably to a cooperative mechanism as previously reported in the literature [50].

### 2.7. Effect of the Sensitizers

In order to select the minimal loading of sensitizer able to produce a significant amount of formic acid, three different quantities of CuPc have been supported on TiO_2_. Typically, porphyrin or phtalocyanine molecules offer enormous potential as the light harvesting components of dye-sensitised TiO_2_ hybrid systems. These are amongst the most used dyes tested for sensitisation of inorganic semiconducting oxides improving the efficiency and thus the determining fundamental electron transfer steps, from charge photogeneration to recombination.

As shown in Table 5, 6.65 µmol was the amount of CuPc which gave a production of formic acid significantly higher than the bare TiO_2_. Higher quantity of sensitizer did not give rise to a significant increase of formic acid production. Notably a high quantity of sensitizer could cover the TiO_2_ surface hindering its photoexcitation. Other types of sensitizers (ZnPp-TiO_2_, ZnPc-TiO_2_ and H_2_Pp-TiO_2_) showed negligible photoactivity in comparison to CuPc, and for this reason the results are not reported for the sake of brevity.

**Table 5:** Effect of sensitizer’s loading on formic acid production efficiency at pH = 3.

Catalyst ^a^	µmol(CuPc)/gTiO_2_	mg/L *(g_cat_)^−1^ ^b^	μmol/g_cat_
TiO_2_		120.5	131.0
TiO_2_-CuPc	4.5	64.3	69.9
TiO_2_-CuPc	6.65	191.8	208.5
TiO_2_-CuPc	8.5	211.0	229.4

^a^ Quantity of catalyst = 0.050 g; ^b^ Irradiation time 8 h.

In Table 6 only the H_2_Pp and CuPp samples are reported. They were shown to be efficient for the photodegradation of 4-NP [38].

**Table 6:** Comparison between the photoactivity of phthalocyanine/TiO_2_ and porphyrin/TiO_2_ samples at pH = 3.

Catalyst ^a,b^	mg/L *(g_cat_)^−1^ ^c^	μmol/g_cat_
TiO_2_-CuPc	191.8	208.5
TiO_2_-H_2_Pc	120.5	75.0
TiO_2_-CuPp	30.0	26.5
TiO_2_-H_2_Pp	48.0	32.6
TiO_2_	120.5	131.0

^a^ Quantity of sensitizer = 6.65 μmol/g TiO_2_; ^b^ Quantity of catalyst = 0.050 g; ^c^ Irradiation time 8 h.

Nevertheless, differently from what observed in the photooxidation of 4-NP, TiO_2_-CuPp and TiO_2_-H_2_Pp showed a scarce photoactivity for CO_2_ photoreduction to formic acid. This behaviour might be ascribed to the energy of their HOMO and LUMO.

**Figure 5:**
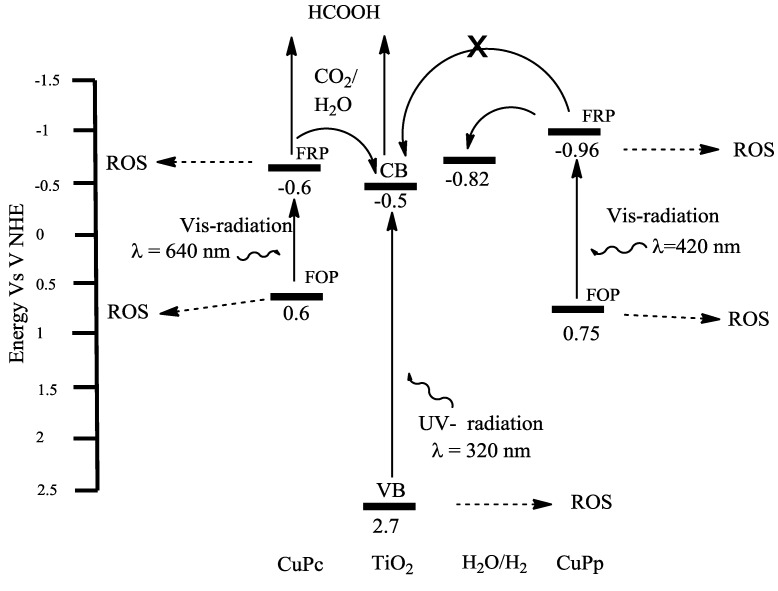
Representation of energy level of TiO_2_, CuPp, CuPc and H_2_O/H_2_ *vs.* NHE.

As reported in literature [51] the First Oxidation Potential (FOP) and the First Reduction Potential (FRP) (equivalent respectively to HOMO and LUMO) of some CuPp samples are about 0.75 and −0.96 V. The above figures could be subjected to slight fluctuations, depending on the solvent in which the measurements are carried out. The conduction band (CB) and the valence band (VB) of TiO_2_ are −0.5 and 2.7 V, respectively. FOP and FRP of CuPp together with the conduction band (CB) and the Valence band (VB) of TiO_2_ are represented in Figure 5.

It can be hypothesized that the negligible activity of CuPp might be caused by the presence of the redox potential of the couple H_2_O/H_2_ between the FRP of CuPp and the CB of TiO_2_. The electron transfer between the sensitizer and the semiconductor, necessary for the occurrence of the photoreduction, could be prevented, whereas reaction (7) can occur:

2 H_2_O + 2 e^−^→ H_2(g)_ + 2 OH^−^ (E_0_ = −0.82 V *vs.* NHE) (7)

The reason why the same composite (CuPp) works efficiently in photodegradation of 4-NP depends on the role of the positive holes. Formation of Reactive Oxygen Species (ROS), like the radical species, in fact, can occur as shown in the following equations:

OH^−^ + *h^+^*_VB_ → ^•^OH (8)

H_2_O + *h^+^*_VB_ → ^•^OH + H^+^ (9)

H_2_O_2_ + *h^+^*_VB_ → HO_2_^•^ + H^+^ (10)

HO_2_^•^ + *h^+^*_VB_ → O_2_ + H^+^ (11)

To our knowledge the values of FRP and FOP of Cu(II) tetrakis[4-(2,4-bis-(1,1-dimethylpropyl)phenoxy)]phthalocyanine are not known; however, these potentials could be considered similar to that measured for the structurally analogous Cu(II) tetra-t-butylphthalocyanine compound [52]. With this assumption, being the FRP of CuPc lower than the potential of water reduction, it could be reasonable to consider the reaction (12) less favored than the CO_2_ photoreduction (see Equation (6)):

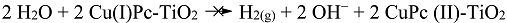
(12)

The possibility to modulate the energy level of porphyrins might prove this hypothesis.

### 2.8. Other Mechanistic Aspects

TiO_2_ has been selected as the photocatalyst to be sensitized because many studies have been reported its effectiveness for the photocatalytic reduction of CO_2_ [3,23,26,27,28,29,53]. Previous papers on photooxidation reactions report the classical mechanism involving the photoexcitation of the dye molecule used as sensitizer under visible light and the subsequent injection of electrons into the conduction band (CB) of TiO_2_ [54,55].

Depending on the experimental conditions used, different reaction pathways can be hypothesized. For example, in the presence of O_2_ into the suspensions the injected electrons (e^−^_CB_) can interact forming the superoxide anion radicals (O_2_^•−^) which is the precursor of ^•^OH and ^•^O_2_H radical species [56]. The latter are reported to have a key role in the photooxidation of many hazardous chemical compounds (like 4-NP) (Equations (13–14)) [38,57,58,59]:

HO_2_^•^ + 4-NP → oxidation products (13)

HO^•^ + 4-NP → oxidation products (14)

In our case, although no oxygen was purging the reacting system, 62 μmol/gr (1.98 mg/L) of molecular oxygen *per gram* of catalyst were detected. The presence of ubiquitous O_2_ was determined by irradiating 8 h at pH = 3 with SANOLUX and RADIUM Xe-Halogen lamps an aqueous suspension (100 mL) of TiO_2_-CuPc sample (0.100 g) containing the optimum quantity of sensitizer (6.65 μmol/g TiO_2_). Photoreduction of CO_2_ by water is only theoretically readily available and inexpensive, due to the high stability of this molecule. Two important species involved in CO_2_ photo-reduction are carbon dioxide anion radical (CO_2_^•−^) and hydrogen radical (H•).

However, for both photoreduction and photodegradation, the key step is the formation of separated charge (hole and electron) on the surface of TiO_2_ (Equation (15)):

TiO_2_ + hν → TiO_2_ (h^+^ + e^−^) (15)

**Figure 6:**
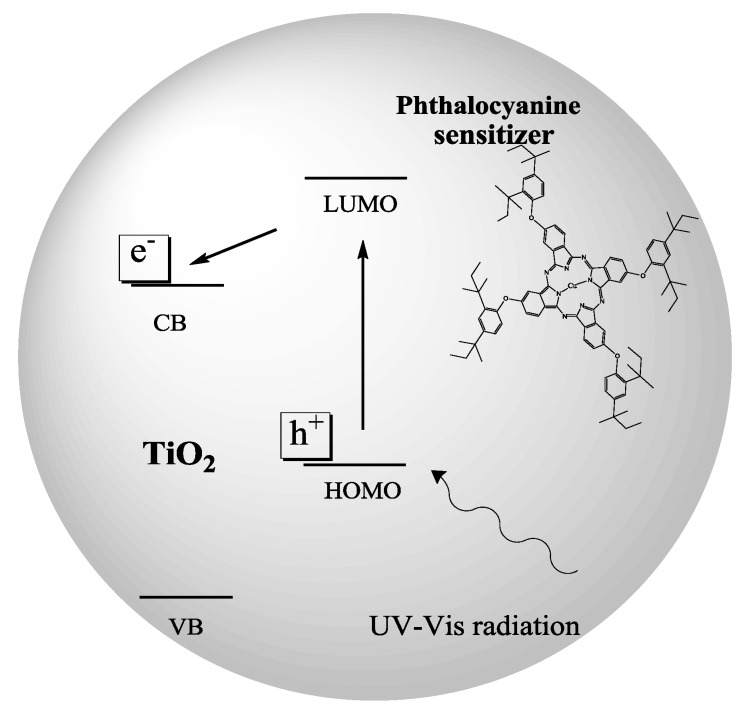
Representation of charge separation enhancement by the use of sensitizer.

The photoinduced process of charge separation promoted by UV-Vis radiation and reported in Figure 6 could be enhanced by the presence of a sensitizer.

**Figure 7:**
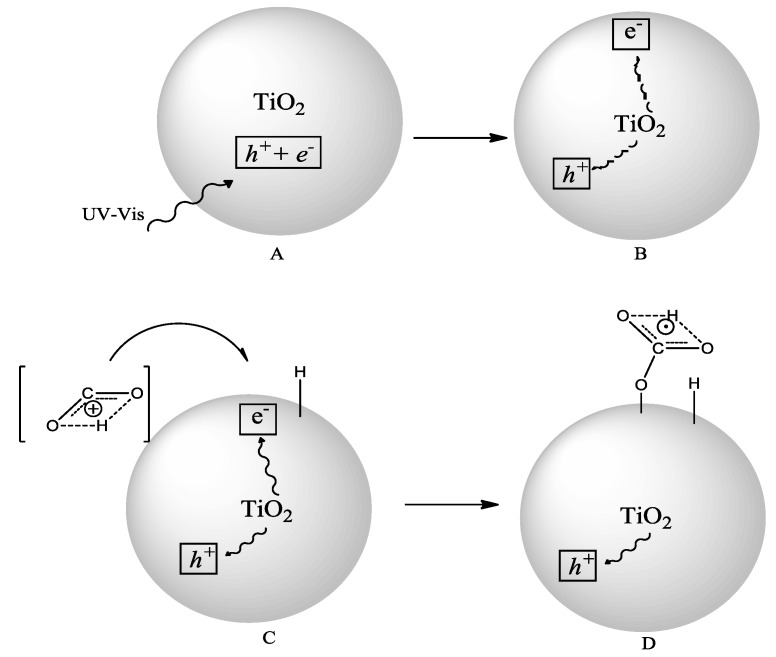
Hypothesized mechanism for the formation of HCOOH on the surface of TiO_2_.

By taking into account the results of the present study and the literature data [54,60] the formation of formic acid by irradiation of TiO_2_ powders can be tentatively explained by the following reaction steps (Figure 7).

Both in the presence and in the absence of sensitizer, once the charges are separated (A), electrons responsible for the photoreduction could migrate on the surface (B) or recombine with holes. Protonated CO_2_can be adsorbed onto the surface of TiO_2_ where it can be reduced to the radical anion CO_2_^•−^(C), to which a close hydroxyl group donates a hydrogen (D), then the formed species can be reduced once again and finally the acid pH favors the desorption of formic acid. At the same time the holes can migrate on the surface where they could react with hole scavengers or interfere with CO_2_^•−^.

The formation of CO_2_^•−^, previously reported as a bent radical anion on a Rh/TiO_2_ composite [60,61], is probably the first step as the aqueous solvent could partially stabilize the species. The radicals were detected near a g value of 2.00 by ESR measurements [23]. Indeed when irradiation of the TiO_2_ powders was performed in highly pure nitrogen medium in the absence of CO_2_, this signal was not observed. The mechanism proposed is alternative to the bidentate linking of CO_2_ to TiO_2_ since no dimerization of reaction intermediates took place during the protonation and formation of oxalic acid was not observed.

## 3. Experimental Section

### 3.1. Materials and Methods

Commercial phosphoric acid 85% (H_3_PO_4_) and sodium hydroxide (NaOH) (purchased by Carlo Erba, Milan, Italy) were used for the preparation of the solutions. Titanium dioxide (pure anatase phase, specific surface area 8 m^2^/g) was kindly provided by Tioxide Huntsman (Scarlino, Italy). CO_2_ purity was 99.999%. Formic acid, 2,4-bis-(1,1-dimethylpropyl)phenol, 4-nitrophthalonitrile, 1,8-diazabicycl[5.4.0]undec-7-ene (DBU) 4-*tert*-butylbenzaldehyde, pyrrole, 2,3-dichloro-5,6-dicyano-1,4-benzoquinone and BF_3_·OEt_2_, were purchased from Sigma-Aldrich (Steinheim, Germany) and were used without purification.

Formic acid analysis was performed by an Ion Chromatograph (IC) Dionex™ DX120 system (Thermo Fisher Scientific Inc. Wyman Street Waltham, MA, USA) equipped with an Ion Pac ICE-AS6 Analytical Column; heptafluorobutyric acid 0.8 mM (Sigma-Aldrich) was used as eluent (flow 1.00 mL/min) and tetrabutylammonium hydroxide 5.0 mM (Sigma-Aldrich) as regenerant (flow 4.5 mL/min). Formic acid 99% (Sigma Aldrich) was employed as standard for building IC calibration curves. The analyses were carried out using a sample loop of 25 µL, with a column pressure less than 640 PSI and a background of 35–40 µS. UV spectra of sensitizers were obtained by using a UV 2401PC spectrometer (Shimadzu Italia S.r.l., Milano, Italy) after dissolving the samples in chloroform (CHCl_3_). Molecular oxygen analysis was performed using a polarographic dissolved oxygen probes Orion 0.86020A (Thermo Fisher Scientific Inc., Wyman Street Waltham, MA, USA). Analysis of labelled formic acid was performed by GCMS-QP2010 Ultra Shimadzu in splitless and SIM mode, using a MS 5 (30 m × 0.25 μm ID) capillary column (40 °C × 10 min, tr 1.82 min). An authentic sample of formic acid ^13^C 95 wt. % in H_2_O, 99 atom % ^13^C (Sigma Aldrich) was used as standard. Commercial sodium bicarbonate ^13^C 98 atom % ^13^C (Sigma Aldrich) was used as source of ^13^CO_2_. 4-[2,4-bis-(1,1-Dimethyl-propyl)phenoxy]phthalonitrile (DPP) was prepared following a procedure reported in the literature [37]. Cu(II)[(5,10,15,20-tetra(4-tertbutylphenyl)] porphyrin (CuPp) and [5,10,15,20-tetra(4-*tert*-butylphenyl)] porphyrin (H_2_Pp) were synthesized using a previously reported procedure [38].

### 3.2. Synthesis of the Cu(II) Tetrakis[4-(2,4-bis-(1,1-dimethylpropyl)phenoxy)]phthalocyanine (CuPc)

**Scheme 1:**
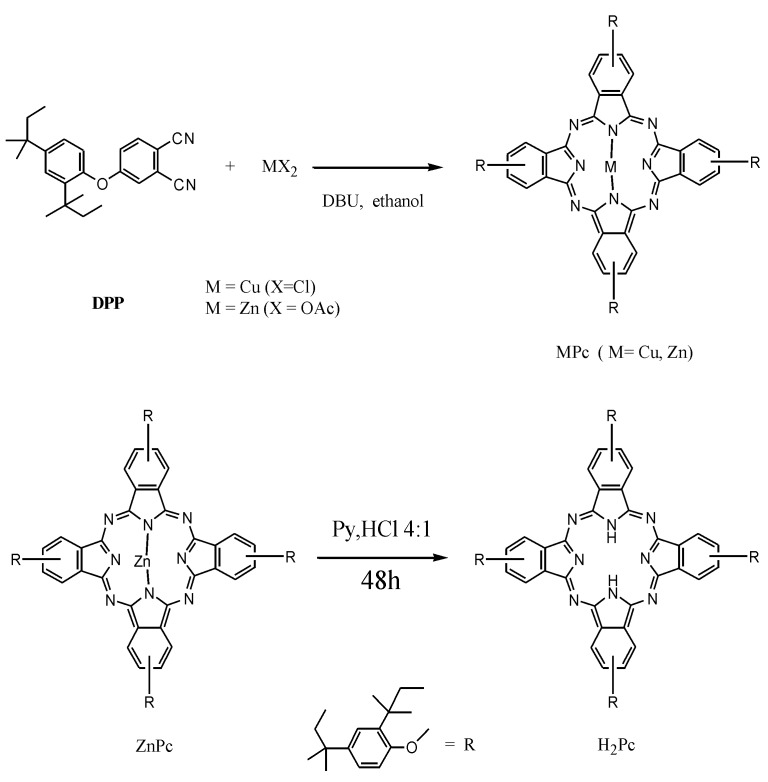
Synthesis of MPcs (M = Cu, Zn) and H_2_Pc.

The CuPc samples were synthesized according to a previously reported procedure [37] and shown in the Scheme 1. Selected spectroscopic data consistent with the structure of CuPc are the following ones: UV-VIS (CHCl_3_) λ_max_: 686, 617, 391, 339, 287 nm.

### 3.3. Synthesis of the Zn(II) Tetrakis[4-(2,4-bis-(1,1-dimethylpropyl)phenoxy)]phthalocyanine *(ZnPc)*

A solution of DPP (300 mg, 0.83 mmol), 1,8-diazabicyclo [5.4.0] undec-7-ene (DBU) (124 mg, 0.81 mmol), and Zn(OAc)_2_ (33 mg, 0.25 mmol) in absolute ethanol (2.5 mL, previously distilled from Mg) was refluxed for 24 h under N_2_ atmosphere. The organic layer enabled the separation of a blue-green solid, which was purified by chromatography (silica, ethyl acetate/hexane 1:9 *v*/*v*) and gave a mixture of isomers of the Zn(II)-tetrakis[4-(2,4-bis-(1,1-dimethyl-propyl)-phenoxy)]phthalocyanine (ZnPc) in 50% yield. The structure was consistent with the following characterization: UV–Vis (CHCl_3_) *λ*_max_: 679, 615, 347, 284.5, 248.

### 3.4. Synthesis of the Tetrakis[4-(2,4-bis-(1,1-dimethylpropyl)phenoxy)]phthalocyanine *(H_2_Pc)*

The tetrakis[4-(2,4-bis-(1,1-dimethylpropyl)phenoxy)]phthalocyanine (H_2_Pc) was prepared by following a procedure reported in literature [62]. ZnPc (126 mg, 0.08 mmol) was dissolved in 10 mL of pyridine and HCl (in molar ratio 4:1). The solution was continuously stirred for 36 h at 120 °C and monitored by TLC (thin-layer chromatography). The reaction mixture was extracted by CH_2_Cl_2_ and the crude product of the reaction, obtained after evaporation of the solvent, was purified by chromatography (silica, ethyl acetate/hexane 0.5:9.5 *v*/*v*). A dark green solid (H_2_Pc) was recovered in 40% yield.

### 3.5. Preparation of TiO_2_-CuPc, TiO_2_-H_2_Pc, TiO_2_-CuPp, TiO_2_-H_2_Pp and TiO_2_-ZnPc Samples

The loaded samples used as photocatalysts for the photoreactivity experiments were prepared by impregnating TiO_2_ (Tioxide, anatase phase) with various amounts of CuPp (6.65 μmol/g TiO_2_); H_2_Pp (6.65 and μmol/g TiO_2_); CuPc (4.5, 6.65 and 8.5 μmol/g TiO_2_); H_2_Pc (6.65 μmol/g TiO_2_) and ZnPc (6.65 μmol/g TiO_2_). The sensitizers were dissolved in 5 mL of CH_2_Cl_2_ and 500 mg of finely ground TiO_2_ (previously sonicated for 30 min) were added to this solution. The mixture was stirred for 6 h and the solvent was removed under vacuum. The code used for the samples is the following: CuPp and CuPc indicate the copper porphyrin and the copper phthalocyanine, H_2_Pp and H_2_Pc the metal-free porphyrin and the metal free phthalocyanine and ZnPc the zinc phthalocyanine. For instance 6.65-TiO_2_–H_2_Pc represents the sample prepared by using 6.65 μmol of metal free phthalocyanine used to impregnate 1 g of TiO_2_ (anatase).

### 3.6. Photoreactivity Experiments

**Figure 8:**
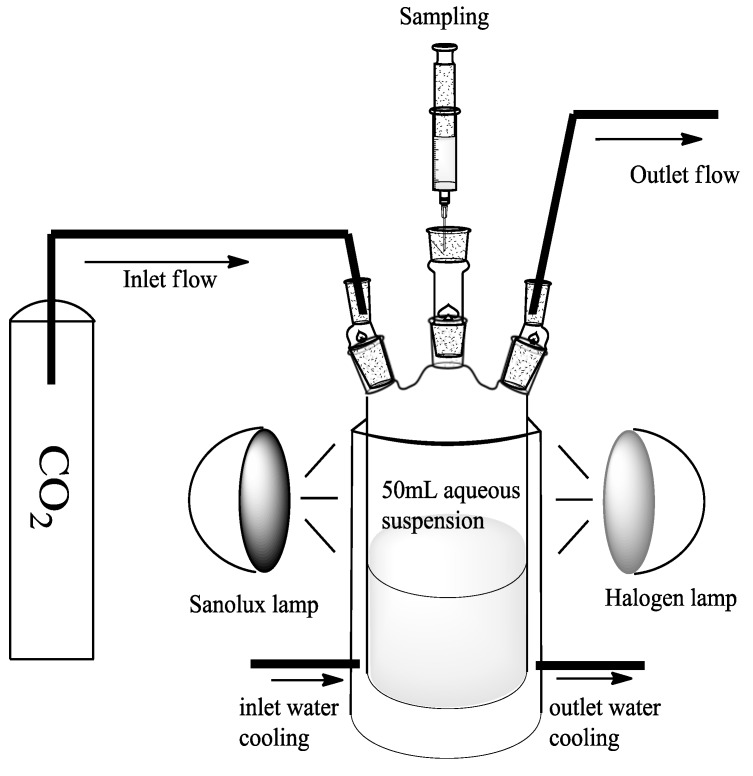
Experimental set-up.

The set-up used for the photocatalytic experiments, reported in Figure 8, consisted of a three necked Pyrex batch photoreactor of cylindrical shape containing 50 mL of aqueous suspension at various pH values. The photoreactor was provided with a jacket for cooling water circulation and ports in its upper section for the inlet and outlet of gases, for sampling and for pH and temperature measurements. A HRC UV-VIS lamp 300 W (Vitalux, OSRAM, Milano, Italy) and Xe-Halogen lamp 400 W (Radium, Wipperfurth, Germany) were placed in proximity (5–6 cm) of the reactor.

Two different processes (indicated as A or B) were carried out in order to reduce CO_2_ produced from different sources.

#### 3.6.1. Process A

CO_2_ was bubbled into the suspensions for approximately 0.5 h before switching on the lamps to saturate the reaction mixture. The amount of catalyst used for the optimization of the reaction was between 50 and 300 mg, whereas that to study the effect of the pH, the macrocycle, the metal and the quantity of sensitizers on the formation of formic acid was 50 mg. The initial pH, except in the basic (0.1 M NaOH) and neutral solution, was adjusted to 3 by addition of H_3_PO_4_. The reactor was tightly sealed during the reaction, stirred continuously by a magnetic bar to prevent catalyst sedimentation and the temperature inside the reactor was held at approximately 25 °C, due to a continuous circulation of water in the jacket around the photoreactor. The photoreactivity runs lasted 8.0 h. Samples of 2.5 mL volume were extracted from the suspension at the end of reaction and filtered through Teflon syringe filters (1.0 mm, Alltech Associates). The quantitative determination of formic acid was performed by measuring its conductivity with the Dionex DX120 Ion Chromatograph.

#### 3.6.2. Process B

NaHCO_3_ solid (150 mg, 1.76 mmol) was mixed with 0.050 g of TiO_2_-CuPc catalyst (6.65 μmol/g TiO_2_) and afterwards 15 mL of aqueous phosphoric acid solution at pH = 3 were added under vigorous stirring in 15 min. Gaseous CO_2_ developed under these conditions. The resulting mixture was allowed to react 8 h at 20–25 °C. The irradiation conditions were the same of those used for the process A. The same procedure was brought using NaHCO_3_^13^C labelled (150 mg, 1.76 mmol). Samples of 2.5 mL volume were extracted from the suspension at the end of reaction and filtered through Teflon syringe filters (1.0 mm, Alltech Associates, Milano, Italy). The qualitative determination of formic acid was performed by GC/MS working in the selected ion monitoring (SIM) mode.

## 4. Conclusions

In this paper, details of a low-cost, stable, effective catalyst capable of CO_2_ photoreduction with production of formic acid has been described. TiO_2_ (anatase) samples impregnated with functionalized metal phthalocyanines can be considered as a versatile class of photocatalysts that can be successfully used not only for the photodegradation of organic micropollutants in aqueous suspension, but also for the efficient photoreduction of CO_2_ under UV-Vis irradiation. To our knowledge, it is the first time that Cu(II)–phthalocyanine samples are successfully used as sensitizers in photoreduction.

TiO_2_ (anatase) samples impregnated with functionalized copper phthalocyanines results to be 62% more effective compared to bare TiO_2_ in the production of formic acid, an important product for its multiple uses. The design of the composites has been inspired by previous studies focused on the utilization of sensitized powders effective for photodegradation of 4-NP. The same materials were tested for CO_2_ photo-reduction carried out in the absence of O_2_.

Negligible activity was observed with TiO_2_ sensitized porphyrins, although it can be excluded that some types of these species could be effective. Further investigation is necessary to clarify the mechanism based on reduction potential, which, if confirmed, could explain why the porphyrins are less effective in the photoreduction than the phthalocyanines.

More details about the FOP and FOM values of the sensitizers could be important to design TiO_2_ composites having photocatalytic properties oriented towards the production of ROS useful for microorganic pollutants or CO_2_ photoreduction in water phases.

The absence of leaching, the mild conditions, the utilization of visible light, and the environmental sustainability could stimulate the use of other hybrid photocatalysts, effective for photo-oxidation reactions, to reduce CO_2_ to formic acid. It would be finally useful to broaden the present study to other sensitizers supported on TiO_2_ both for the sake of comparison and to better understand some mechanistic aspects.
